# Analysis of clinical outcomes and meiotic segregation modes following preimplantation genetic testing for structural rearrangements using aCGH/NGS in couples with balanced chromosome rearrangement

**DOI:** 10.1002/rmb2.12476

**Published:** 2022-06-29

**Authors:** Tatsuya Nakano, Michiko Ammae, Manabu Satoh, Satoshi Mizuno, Yoshiharu Nakaoka, Yoshiharu Morimoto

**Affiliations:** ^1^ IVF Namba Clinic Osaka City Osaka Japan; ^2^ IVF Osaka Clinic Osaka City Osaka Japan; ^3^ HORAC Grandfront Osaka Clinic Osaka City Osaka Japan

**Keywords:** aCGH/NGS, meiotic segregation mode, PGT‐SR, recurrent miscarriage

## Abstract

**Purpose:**

To retrospectively evaluate the effectiveness of PGT‐SR by array comparative genomic hybridization (aCGH) or next‐generation sequencing (NGS) in preventing recurrent miscarriages.

**Methods:**

Thirty one couples with balanced translocation who underwent 68 PGT‐SR cycles between 2012 and 2020 were evaluated. A total of 242 blastocysts were biopsied for aCGH or NGS. The genetically transferable blastocysts were transferred in the subsequent frozen‐thawed single embryo transfer cycle.

**Results:**

The genetically transferable blastocyst rate was 21.2% (51/241). Thirty five genetically transferable blastocysts were transferred into the uterine cavity. The clinical pregnancy rate was 57.1% (20/35), and the ongoing pregnancy rate was 100.0% (20/20). The incidence of interchromosomal effect (ICE) was influenced by ovarian stimulation protocol, female age, and carrier's gender, but dependent on the types of balanced translocation carriers. Furthermore, there was no significant difference in meiotic segregation modes in ovarian stimulation protocols and carrier's gender. Interestingly, the incidence of adjacent‐1 segregation in ≧40 years group increased significantly compared with <35 years group.

**Conclusions:**

For the first time in Japan, we show the effectiveness of PGT‐SR using aCGH or NGS, which enables comprehensive analysis of chromosomes, in the prevention of recurrent miscarriages. Furthermore, our results may support better genetic counseling of balanced translocation carriers for PGT‐SR cycles.

## 1 INTRODUCTION

Balanced structural chromosomal rearrangements such as reciprocal translocations (RecT), Robertsonian translocations (RobT), and inversions (Inv) are the most frequent chromosomal structural abnormalities. The phenotype of carriers with balanced translocations is normal, occurring in 0.2% of newborns and is found in 1%–5% of recurrent miscarriage couples.^1^, ^2^, ^3^ However, they have a high risk of recurrent miscarriages or birth defects due to chromosomally abnormal embryos from the unbalanced gametes produced.^4^, ^5^, ^6^, ^7^ During meiosis of RecT, the translocated chromosomes and their normal homologs form quadrivalent chromosomes and cause adjacent‐1, adjacent‐2, 3:1, or 4:0 segregation.^8^ Whereas, RobT has chromosomal rearrangements that result from the fusion of the entire long arms of two acrocentric chromosomes resulting in a trivalent chromosome that during meiosis results in chromosomal abnormality.^9^ These abnormal gametes increase the risk of miscarriage, especially in first‐trimester abortions. In general, balanced translocation carriers have a high risk of recurrent miscarriage, approximately 50%–80% for RecT carriers and 50% for RobT.^3^, ^10^, ^11^

Preimplantation genetic testing for structural rearrangements (PGT‐SR) is an effective method of diagnosis for recurrent miscarriages in balanced translocation carriers. This method analyzes chromosomes using some of the cells of the preimplantation embryo and allows the selection of balanced/normal euploid embryos for embryo transfer.^12^, ^13^ Thus, it improves pregnancy outcomes in couples with balanced translocations, reducing the time to achieve a healthy live birth from 4–6 years to less than 4 months and decreasing the miscarriage rate to less than 15% in RecT carriers.^12^, ^13^ Previously, fluorescence in situ hybridization (FISH) was widely used for PGT‐SR to distinguish balanced embryos from unbalanced ones.^14^, ^15^, ^16^ FISH uses a probe that fluorescently labels a specific region of a chromosome to analyze the presence or absence of that particular region. Therefore, it is necessary to prepare several probes corresponding to chromosomal structural abnormalities for each translocation carrier.^17^ In addition, many steps of the procedure depend on the technician's skill in preparing the specimen, which affects the diagnostic accuracy.^18^ On the other hand, the clinical application of new technologies such as comprehensive chromosomal screening using array comparative genomic hybridization (aCGH) and next‐generation sequencing (NGS) has been shown to improve the clinical outcomes of PGT‐SR.^19^, ^20^, ^21^, ^22^ These new methods require whole genome amplification (WGA). They improve diagnostic accuracy because many processes are mechanized. Therefore, these have been now commonly used in PGT‐SR chromosome analysis and recently it has been in use in Japan too. However, in Japan, there are only a few reports of the analysis by PGT‐SR using these methods and clinical results are still unknown.^23^

The chromosomal segregation on the spindle alignment in first meiosis is critical. In balanced translocation carriers, the chromosomes involved in the rearrangement would have a detrimental effect on the segregation of the structurally normal chromosomes. This is defined as the interchromosomal effect (ICE).^24^ Since comprehensive chromosomal screening in PGT‐SR is done now by aCGH or NGS, ICE is in focus. So far, translocation carriers with the acrocentric chromosome or telomere region have been reported to have a high chromosomal abnormality rate.^25^ RobT carriers are shown to have more impact on ICE than RecT carriers, albeit in some contradictory reports.^26^, ^27^, ^28^ However, it is still unclear how ovarian stimulation, female age at oocyte retrieval, carrier gender, and ICE impact chromosomal abnormalities during meiosis in translocation carriers.

This study aims to evaluate the efficacy of PGT‐SR using aCGH or NGS in recurrent miscarriage prevention. In addition, we assessed the effects of female age, carrier gender, ovarian stimulation, etc., on chromosomal segregation during rearrangement in first meiosis.

## 2 MATERIALS AND METHODS

### 2.1 Study population

In this retrospective analysis, PGT‐SR results were reviewed for 68 oocyte retrieval cycles of 31 couples from February 2012 to April 2020. Clinical indications for PGT‐SR were reciprocal translocation in 26 couples, Robertsonian translocation in 4 couples, and pericentric inversion in 1 couple, with a previous clinical history of recurrent miscarriages in natural conception or IVF pregnancy (Table S1).

All patients underwent a systematic examination, including hysterosalpingography, diagnostic tests for antiphospholipid syndrome (APS), including screening for lupus anticoagulant by activated partial thromboplastin time (aPTT) and dilute Russell's viper venom time and (βeta2 glycoprotein I‐dependent) anticardiolipin antibody, and blood tests for hypothyroidism and diabetes mellitus, before a subsequent pregnancy. The results of these diagnoses are shown in Table S1.

### 2.2 Ovarian stimulation

Patients were treated with controlled ovarian stimulation based on their medical history as follows. All protocols performed controlled ovarian hyperstimulation with recombinant follicle‐stimulating hormone (r‐FSH; (Gonal F®; Merck Serono) and human menopausal gonadotropin (HMG; Ferring Pharmaceuticals). In the GnRH agonist cycle, patients started oral contraceptive pills (1 mg of norethisterone and 0.05 mg of mestranol; Aska Pharmaceutical) on day 14 of the previous cycle for 10 days. After that, GnRH agonist (600 μg/day, Suprecur® nasal solution 0.15%; Mochida Pharmaceutical) was administered from day 21 of the previous cycle until ovulation induction. On the third day of the stimulation cycle, r‐FSH in the range of 150–450 IU was administered for 4 days, followed by continuous administration of HMG in the range of 150–450 IU until ovulation induction. In the GnRH antagonist cycle, GnRH antagonist was administered daily after the leading follicle reached 13–14 mm in diameter. In the mild stimulation cycle, Clomiphene (Clomid®; Fuji Pharma Co.) or letrozole (Femara Tablets® 2.5 mg; Novartis) was administered for 5 days from day 3, followed by continuous administration of HMG in the range of 150–450 IU until ovulation induction. Ovulation induction was performed by human chorionic gonadotropin (hCG) administration when the leading follicle reached 18 mm in diameter. Transvaginal follicle aspiration was performed 36 h post hCG injection.

### 2.3 Intracytoplasmic sperm injection, blastocyst culture, and biopsy

After oocyte retrieval, intracytoplasmic sperm injection (ICSI) was performed on metaphase II oocytes in all cases. 16–20 h after ICSI, those with 2 pronuclei were considered as normally fertilized oocytes and cultured to blastocysts. The embryos were cultured in sequential media (SAGE 1‐step; CooperSurgical) at 37°C under 6.0% CO2, 5.0% O2 and 89.0% N2. Blastocysts were graded according to the Gardner blastocyst morphological scoring system.^29^ Blastocysts with ≧3BB grades (3, 4, 5, 6, AA, AB, BA, and BB) were defined as good‐quality blastocysts and those with C grade were defined as poor‐quality blastocysts. On day 5 or 6 of the culture, the zona pellucida of the blastocysts was opened using a laser system (ZIROS‐tk laser system; Hamilton Thorne Biosciences), and a recovery culture was performed until about 5–10 of the trophectoderm (TE) cells escaped from the zona pellucida. The cells were aspirated into a biopsy pipette of 25 μm internal diameter and biopsied (cell membrane breaking) with a laser. The biopsied TE cells were transferred into PCR tubes with 1% polyvinylpyrrolidone/phosphate‐buffered saline and stored at −20°C until WGA was performed. Furthermore, the biopsied blastocysts were immediately cryopreserved by vitrification method using Cryotop (Kitazato Corporation). Blastocysts with an evaluation grade of ≧3 BC were used for the biopsy and those with the inner cell mass of grade C were excluded.

### 2.4 Whole genome amplification, aCGH, and NGS

According to the manufacturer's protocol, the biopsied TE cells were lysed and the whole genome was amplified using a SurePlex WGA Kit (Illumina). For chromosomal analysis with aCGH, the WGA products and control DNA were labeled with Cy3 and Cy5 fluorophores according to the manufacturer's instructions and hybridized on 24sure + arrays (Illumina). For chromosomal analysis with NGS, the WGA products were used for library construction with the VeriSeq DNA Library. According to the manufacturer's protocol, NGS was performed on a MiSeqDx instrument (Illumina) using MiSeqDx Universal Kit 3v (Illumina). All results were analyzed using BlueFuse Multi analysis software (Illumina) for chromatin loss or gain across all 24 chromosomes.

### 2.5 Vitrified normal/balanced blastocyst transfer

During the embryo transfer cycle, the endometrium was prepared by increasing doses of oral estradiol valerate (Progynova®; Bayer Schering Pharma) from 1 to 4 mg for 2 weeks along with the administration of GnRH agonist. After ultrasonographical confirmation that the endometrium was thicker than 8 mm, 6 mg/day of chlormadinone acetate (Lutoral®; Shionogi & Co.) was started. Blastocyst transfer was carried out on the 5th day of chlormadinone acetate administration. On the day of the transfer, vitrified normal/balanced blastocysts were thawed using Kitazato warming solution (Kitazato Corporation). After warming and dilution, blastocysts were cultured in a SAGE 1‐step medium for 1–2 h before transferring into the uterine cavity. Progesterone (Progeston depot® 125 mg; Fuji Pharma Co.) was administered intramuscularly on the day of embryo transfer, and daily doses of 3 mg estradiol valerate and 6 mg chlormadinone acetate were maintained until the pregnancy test. When pregnancy was confirmed, estradiol (2.88 mg every 2 days, Estradna®; Hisamitsu) was administered transcutaneously and progesterone (400 mg/day, Utrogestan® 200 mg; Ferring Pharmaceuticals) was administered transvaginally until 9 weeks of gestation. Moreover, APS patients were excluded from the embryo transfer results.

The establishment of pregnancy was determined at around 2 weeks after embryo transfer by the blood hCG concentration of ≧100 mIU/ml. Clinical pregnancy was determined at around 3 weeks after embryo transfer by detecting a single intrauterine gestational sac by transvaginal ultrasonography. It was considered as an ongoing pregnancy when no miscarriage was observed by 24 weeks.

### 2.6 Statistics

Data are expressed as the mean ± standard deviation (SD). Comparison of means was conducted by one‐way analysis of variance (ANOVA) using Tukey's multiple range test. Comparisons of proportions were evaluated using the chi‐square exact tests. Statistical analyses were performed using StatView version 5.0 (SAS Institute). *p* < 0.05 was considered statistically significant.

## 3 RESULTS

### 3.1 Clinical outcomes of all PGT‐SR cycles

Table 1 summarizes the results of all PGT‐SR cycles. The average age of females and males at oocyte retrieval was 37.0 and 39.2 years. Nine hundred twenty one921 cumulus‐oocyte complexes (COCs) were retrieved, and ICSI was performed on 727 MII oocytes. Of these, 81.3% (591/727) confirmed normal fertilization having 2 pronuclei (2PN) and 62.6% (370/591) developed into blastocysts. A total of 242 blastocysts were biopsied and 16 of them were re‐biopsied as the DNA amplification by WGA was unsuccessful. Despite re‐biopsy, DNA amplification could not be confirmed in one of the blastocysts. Of the 241 WGA successful blastocysts, the proportion of genetically transferable blastocysts (i.e., euploid, balanced, or mosaic; mosaic was defined as from 30% or more to less than 70% aneuploidy) was 21.2% (51/241). Thirty five genetically transferable blastocysts were transferred into the uterine cavity. The clinical pregnancy rate was 57.1% (20/35) and the ongoing pregnancy rate was 100.0% (20/20). In addition, the ongoing pregnancy rate per patient who underwent PGT‐SR was 57.1% (16/28).

**TABLE 1:** The characteristics and clinical outcomes of patients who underwent preimplantation genetic testing for structural rearrangements (PGT‐SR)

Number of patients	31
Number of OR cycles	68
Female age at OR (years)	37.0 ± 4.5
Male age at OR (years)	39.2 ± 5.2
Number of COCs per cycle	13.5 ± 9.7
Fertilization (2PN) rate (%) (/Matured oocyte)	81.3 (591/727)
Blastocyst formation rate (%) (/Fertilized oocytes)	62.6 (370/591)
Biopsied blastocyst rate (%) (/Blastocysts)	65.4 (242/370)
Re‐biopsied blastocyst rate (%) /Biopsied blastocysts)	6.6 (16/242)
Informative result rate (%) (/Biopsied blastocysts)	99.6 (241/242)
Genetically transferable blastocyst rate (%) (/Analyzed blastocysts)	21.2 (51/241)
Number of ET cycles	35
Female age at ET (year)	35.9 ± 3.1
Morphologically good blastocyst rate (%) (/Transferred blastocysts)	71.4 (25/35)
Clinical pregnancies (%) (/ET cycles)	57.1 (20/35)
Ongoing pregnancy rate (%) (/Clinical pregnancies)	100.0 (20/20)
Ongoing pregnancy rate per patient (%) (/Patients who underwent PGT‐SR)	57.1 (16/28)

*Note:* Values for each parameter are presented as the mean ± standard deviation (SD).

Abbreviations: COCs, cumulus‐oocyte complex; ET, embryo transfer; OR, oocyte retrieval; 2PN, 2 pronuclei.

### 3.2 Comparison of stimulation protocol

Table 2 shows a comparison of the characteristics and clinical results of couples who were stimulated by GnRH agonist, GnRH antagonist, and mild stimulation protocols. There was no significant difference in the average age of females and males at oocyte retrieval between GnRH agonist (35.8 and 37.9) and GnRH antagonist (35.7 and 37.2), but it was significantly increased in the mild stimulation group (40.1 and 43.4).

**TABLE 2:** The characteristics and clinical outcomes of the three stimulation protocols

	GnRH agonist	GnRH antagonist	Mild stimulation
Number of OR cycles	23	25	22
Female age at OR (years)	35.8 ± 3.3^a^	35.7 ± 2.8^a^	40.1 ± 5.3^b^
Male age at OR (years)	37.9 ± 5.8^a^	37.2 ± 4.3^a^	43.4 ± 4.5^b^
Number of COCs per cycle	17.7 ± 8.9^a^	18.3 ± 9.6^a^	4.3 ± 2.4^b^
Fertilization (2PN) rate (%) (/Matured oocyte)	83.0 (254/306)	79.7 (275/345)	81.6 (62/76)
Blastocyst formation rate (%) (/Fertilized oocytes)	60.2 (153/254)	62.6 (172/275)	72.6 (45/62)
Morphologically good blastocyst rate (%) (/Fertilized oocytes)	30.3 (77/254)	28.0 (77/275)	16.1 (10/62)
Biopsied blastocyst rate (%) (/Blastocysts)	68.0 (104/153)^a^	68.6 (118/172)^a^	44.4 (20/45)^b^
Genetically transferable blastocyst rate (%) (/Analyzed blastocysts)	22.3 (23/103)^a^	22.9 (27/118)^a^	5.0 (1/20)^b^
Number of ET cycles	15	19	1
Female age at ET (year)	36.0 ± 3.3	35.9 ± 2.4	34.0 ± 0.0
Morphologically good blastocyst rate (%) (/Transferred blastocysts)	86.7 (13/15)	57.9 (11/19)	100.0 (1/1)
Clinical pregnancies (%) (/ET cycles)	80.0 (12/15)	36.8 (7/19)	100.0 (1/1)
Ongoing pregnancy rate (%) (/Clinical pregnancies)	100.0 (12/12)	100.0 (7/7)	100.0 (1/1)

*Note*: Values for each parameter are presented as the mean ± standard deviation (SD).

Different letters indicate significant differences (*p* < 0.05).

Abbreviations: COCs, cumulus‐oocyte complex; ET, embryo transfer; GnRH, gonadotropin‐releasing hormone; OR, oocyte retrieval; 2PN, 2 pronuclei.

In the results of oocyte retrieval and embryo culture, there was no difference in the mean number of retrieved COCs between GnRH agonist (17.7) and GnRH antagonist (18.3), but it was significantly decreased in the mild stimulation group (4.3). In addition, there was no difference in the normal fertilization rate, the blastocyst formation rate, and morphologically good blastocyst rate among the protocols. There were no significant differences between GnRH agonist (68.0%) and GnRH antagonist (68.6%) in the biopsied blastocyst rates (blastocysts that could be biopsied), but they were significantly decreased in the mild stimulation group (44.4%). Moreover, there was no difference in the proportion of genetically transferable blastocysts between GnRH agonist (22.3%) and GnRH antagonist (22.9%), but it was significantly decreased in the mild stimulation group (5.0%).

In the results of embryo transfer, there were no significant differences in the female age at embryo transfer, morphologically good blastocyst rate, clinical pregnancy rate, and ongoing pregnancy rate between GnRH agonist and GnRH antagonist. On the other hand, as there was only one cycle for mild stimulation protocol, it was not possible to compare by statistical analysis for embryo transfer. Nevertheless, one genetically transferable blastocyst was obtained, leading to ongoing pregnancies post frozen‐thaw blastocyst transfer.

### 3.3 Comparison of female age at oocyte retrieval

Table 3 shows a comparison of the characteristics and clinical outcomes by the female age at oocyte retrieval, which was divided into three groups: under 35 years old (<35), between 35 to 39 years old (35–39), and 40 years and above (≧40). There was no difference in the mean number of retrieved COCs between <35 years (17.1) and 35–39 years group (15.8), but it significantly decreased in ≧40 years group (5.7). In addition, the normal fertilization rate and blastocyst formation rate in <35 years group (77.0% and 54.6%) were significantly lower than in the 35–39 years group (85.4% and 66.5%), but there was no difference in ≧40 years group (82.8% and 75.3%). Furthermore, there were no significant differences in the morphologically good blastocyst rate and the biopsied blastocyst rate among the groups. However, there was no difference in the rate of genetically transferable blastocysts between<35 years (17.8%) and the 35–39 years groups (29.7%), but no genetically transferable blastocysts were obtained in ≧40 years group.

**TABLE 3:** The characteristics and clinical outcomes by female age at oocyte retrieval

Female age at OR	<35	35–39	≧40
Number of OR cycles	26	24	18
Female age at OR (years)	32.6 ± 1.9^a^	37.6 ± 2.3^b^	41.8 ± 2.2^c^
Male age at OR (years)	34.0 ± 3.1^a^	39.1 ± 4.4^b^	43.7 ± 9.5^c^
Number of COCs per cycle	17.1 ± 12.3^a^	15.8 ± 5.7^a^	5.7 ± 3.7^b^
Fertilization (2PN) rate (%) (/Matured oocyte)	77.0 (251/326)^a^	85.4 (263/308)^b^	82.8 (77/93)^a^
Blastocyst formation rate (%) (/Fertilized oocytes)	54.6 (137/251)^a^	66.5 (175/263)^b^	75.3 (58/77)^a^
Morphologically good blastocyst rate (%) (/Fertilized oocytes)	25.5 (64/251)	30.0 (79/263)	27.3 (21/77)
Biopsied blastocyst rate (%) (/Blastocysts)	66.4 (91/137)	67.4 (118/175)	56.9 (33/58)
Genetically transferable blastocyst rate (%) (/Analyzed blastocysts)	17.8 (16/90)^a^	29.7 (35/118)^a^	0.0 (0/33)^b^
Number of ET cycles	14	21	‐
Female age at ET (year)	33.1 ± 2.2^a^	37.8 ± 1.7^b^	‐
Morphologically good blastocyst rate (%) (/Transferred blastocysts)	50.0 (7/14)	85.7 (18/21)	‐
Clinical pregnancies (%) (/ET cycles)	64.3 (9/14)	52.4 (11/21)	‐
Ongoing pregnancy rate (%) (/Clinical pregnancies)	100.0 (9/9)	100.0 (11/11)	‐

*Note:* Values for each parameter are presented as the mean ± standard deviation (SD).

Different letters indicate significant differences (*p* < 0.05).

Abbreviations: COCs, cumulus‐oocyte complex; ET, embryo transfer; OR, oocyte retrieval; 2PN, 2 pronuclei.

In the results of embryo transfer, although there was a difference in maternal age at embryo transfer between <35 years (33.1) and 35–39 years groups (37.8), there was no difference in morphologically good blastocyst rate, clinical pregnancy rate, and ongoing pregnancy rate.

### 3.4 Comparison of carrier gender

Table 4 shows a comparison of the characteristics and clinical results of female carriers and male carriers. There was no significant difference in the average age of females and males at oocyte retrieval between the female carriers and male carriers. The mean number of retrieved COCs in male carriers (16.6) was significantly higher than in female carriers (11.8). However, the normal fertilization rate, the blastocyst formation rate, and the morphologically good blastocyst rate in male carriers (75.1%, 54.9%, and 22.1%) were significantly lower than in female carriers (86.0%, 67.7%, and 31.5%). There were no significant differences in the biopsied blastocyst rate and the genetically transferable blastocyst rate between female carriers and male carriers. Furthermore, there were no significant differences in the clinical pregnancy rate and ongoing pregnancy rate between the female and male carriers.

**TABLE 4:** The characteristics and clinical outcomes of couples with female carrier vis‐a‐vis male carrier

	Female carrier	Male carrier
Number of patients	22	9
Number of OR cycles	43	25
Female age at OR (years)	36.1 ± 3.4	36.2 ± 6.4
Male age at OR (years)	37.1 ± 5.2	39.3 ± 7.3
Number of COCs per cycle	11.8 ± 8.5^a^	16.6 ± 11.5^b^
Fertilization (2PN) rate (%) (/Matured oocyte)	86.0 (356/414)^a^	75.1 (235/313)^b^
Blastocyst formation rate (%) (/Fertilized oocytes)	67.7 (241/356)^a^	54.9 (129/235)^b^
Morphologically good blastocyst rate (%) (/Fertilized oocytes)	31.5 (112/356)^a^	22.1 (52/235)^b^
Biopsied blastocyst rate (%) (/Blastocysts)	68.1 (164/241)	60.5 (78/129)
Genetically transferable blastocyst rate (%) (/Analyzed blastocysts)	23.3 (38/163)	16.7 (13/78)
Number of ET cycles	26	9
Female age at ET (year)	36.1 ± 2.7	35.4 ± 3.5
Morphologically good blastocyst rate (%) (/Transferred blastocysts)	76.9 (20/26)	55.6 (5/9)
Clinical pregnancies (%) (/ET cycles)	53.8 (14/26)	66.7 (6/9)
Ongoing pregnancy rate (%) (/Clinical pregnancies)	100.0 (14/14)	100.0 (6/6)

*Note:* Values for each parameter are presented as the mean ± standard deviation (SD).

Different letters indicate significant differences (*p* < 0.05).

Abbreviations: COCs, cumulus‐oocyte complex; ET, embryo transfer; OR, oocyte retrieval; 2PN, 2 pronuclei.

### 3.5 Comparison among different types of balanced translocation carriers

Table 5 shows the comparison of the characteristics and clinical results of reciprocal translocation (RecT), Robertsonian translocation (RobT), and pericentric inversion (Inv). There were no significant differences in the average age of females and males at oocyte retrieval between RecT carriers and RobT carriers. However, the mean number of retrieved COCs in RecT carriers (13.1) was significantly lower than in RobT carriers (19.0). In addition, there was no significant difference in the normal fertilization rate, the blastocyst formation rate, the morphologically good blastocyst rate and the biopsied blastocyst rate between RecT carriers and RobT carriers. However, the genetically transferable blastocyst rates in RecT carriers (16.3%) were significantly lower than in RobT carriers (39.5%).

**TABLE 5:** The characteristics and clinical outcomes by type of rearrangement

	Reciprocal translocation	Robertsonian translocation	Pericentric inversion
Number of patients	26	4	1
Number of OR cycles	62	5	1
Female age at OR (years)	37.0 ± 3.9	35.8 ± 2.6	38.1 ± 0.0
Male age at OR (years)	39.2 ± 5.5	38.2 ± 4.7	39.3 ± 0.0
Number of COCs per cycle	13.1 ± 10.0^a^	19.0 ± 3.1^b^	12.0 ± 0.0
Fertilization (2PN) rate (%) (/Matured oocyte)	81.0 (511/631)	83.7 (72/86)	80.0 (8/10)
Blastocyst formation rate (%) (/Fertilized oocytes)	60.9 (311/511)	76.4 (55/72)	50.0 (4/8)
Morphologically good blastocyst rate (%) (/Fertilized oocytes)	27.8 (142/511)	27.8 (20/72)	25.0 (2/8)
Biopsied blastocyst rate (%) (/Blastocysts)	63.3 (197/311)	78.2 (43/55)	50.0 (2/4)
Genetically transferable blastocyst rate (%) (/Analyzed blastocysts)	16.3 (32/196)	39.5 (17/43)^b^	100.0 (2/2)
Number of ET cycles	23	10	2
Female age at ET (year)	35.6 ± 2.8	36.6 ± 2.9	38.5 ± 0.0
Morphologically good blastocyst rate (%) (/Transferred blastocysts)	73.9 (17/23)	60.0 (6/10)	100.0 (2/2)
Clinical pregnancies (%) (/ET cycles)	65.2 (15/23)	50.0 (5/10)	0.0 (0/2)
Ongoing pregnancy rate (%) (/Clinical pregnancies)	100.0 (15/15)	100.0 (5/5)	‐

*Note:* Values for each parameter are presented as the mean ± standard deviation (SD).

Different letters indicate significant differences (*p* < 0.05).

Abbreviations: COCs, cumulus‐oocyte complex; ET, embryo transfer; OR,oocyte retrieval; 2PN, 2 pronuclei.

Furthermore, there were no significant differences in clinical pregnancy rate and ongoing pregnancy rate between RecT carriers and RobT carriers. On the other hand, as there was only one cycle for Inv carrier and insufficient numbers of oocytes retrieved and embryos cultured, it was not possible to compare by statistical analysis in Inv carrier. Although two genetically transferable blastocysts were obtained, they did not lead to pregnancy in frozen‐thaw blastocyst transfer.

### 3.6 Incidence of chromosomal abnormalities

Table 6 shows the analysis of chromosomal abnormalities in blastocysts for all PGT‐SR cycles. Overall, the normal/balanced (referred to as balanced in the tables) blastocyst rate was 21.2%, the unbalanced and euploid (unbalanced) blastocyst rate was 27.4%, the unbalanced and aneuploid (unbalanced + aneuploid) blastocyst rate was 32.4%, and the balanced and aneuploid (aneuploid) blastocyst rate was 19.1%. Furthermore, the proportion of total interchromosomal effect (ICE; unbalanced + aneuploid blastocysts and aneuploid blastocysts) was 51.5%. In this study, chromosome aneuploidy involved in rearrangement is not included in ICE.

**TABLE 6:** Analysis of the incidence of chromosomal abnormalities in preimplantation genetic testing for structural rearrangements (PGT‐SR) cycles

	Balanced blastocyst rate (%) (/Analyzed blastocysts)	Unbalanced blastocyst rate (%) (/Analyzed blastocysts)	Unbalanced + Aneuploid blastocyst rate (%) (/Analyzed blastocysts)	Aneuploid blastocyst rate (%) (/Analyzed blastocysts)	Total ICE rete (%) (Unbalanced + Aneuploid and Aneuploid) (/Analyzed blastocysts)
Total	21.2 (51/241)	27.4 (66/241)	32.4 (78/241)	19.1 (46/241)	51.5 (124/241)
Stimulation method					
GnRH agonist	22.3 (23/103)	28.2 (29/103)	31.1 (32/103)	18.4 (19/103)^a^	49.5 (51/103)^a^
GnRH antagonist	22.9 (27/118)	29.7 (35/118)	31.4 (37/118)	16.1 (19/118)^a^	47.5 (56/118)^a^
Mild stimulation	5.0 (1/20)	10.0 (2/20)	45.0 (9/20)	40.0 (8/20)^b^	85.0 (17/20)^b^
Female age at OR					
<35	17.8.(16/90)^a^	33.3 (30/90)^a^	26.7 (24/90)^a^	22.2 (20/90)^a^	48.9 (44/90)^a^
35–39	29.7 (35/118)^a^	28.0 (33/118)^a^	30.5 (36/118)^a^	11.9 (14/118)^a^	42.4 (50/118)^a^
≧40	0.0 (0/33)^b^	9.1 (3/33)^b^	54.5 (18/33)^b^	36.4 (12/33)^b^	90.9 (30/33)^b^
Carrier gender					
Female	23.3 (38/163)	35.0 (57/163)^a^	28.2 (46/163)	13.5 (22/163)^a^	41.7 (68/163)^a^
Male	16.7 (13/78)	11.5 (9/78)^b^	41.0 (32/78)	30.8 (24/78)^b^	71.8 (56/78)^b^
Type of rearrangement					
Reciprocal translocation	16.3 (32/196)^a^	27.0 (53/196)	36.2 (71/196)^a^	20.4 (40/196)	56.6 (111/196)^a^
Robertsonian translocation	39.5 (17/43)^b^	30.2 (13/43)	16.3 (7/43)^b^	14.0 (6/43)	30.2 (13/43)^b^
Pericentric inversion	100.0 (2/2)	0.0 (0/2)	0.0 (0/2)	0.0 (0/2)	0.0 (0/2)

*Note*: Different letters indicate significant differences (*p* < 0.05).

Abbreviations: GnRH, gonadotropin‐releasing hormone; OR, oocyte retrieval.

There was no significant difference in balanced blastocyst rate, unbalanced blastocyst rate, and unbalanced + aneuploid blastocyst rate among the protocols. However, the aneuploid blastocyst rate in the mild stimulation group (40.0%) was significantly higher than that in the GnRH agonist (18.4%) and GnRH antagonist (16.1%) groups. In addition, there was no difference in the proportion of total ICE between the GnRH agonist (49.5%) and the GnRH antagonist (47.5%) group, but they were significantly increased in the mild stimulation group (85.0%).

The balanced blastocyst and unbalanced blastocyst rate in ≧40 years group (0.0% and 9.1%) were significantly lower than in <35 years (17.8% and 33.3%) and 35–39 years groups (29.7% and 28.0%). Furthermore, unbalanced + aneuploid blastocyst, aneuploid blastocyst, and total ICE rate in ≧40 years group (54.5%, 36.4%, and 90.9%) were significantly higher than in <35 years (26.7%, 22.2%, and 48.9%) and 35–39 years (30.5%, 11.9% and 42.4%) groups.

There was no significant difference in the balanced blastocysts and unbalanced + aneuploid blastocysts rate between the female and male carriers. However, the unbalanced blastocyst rate in female carriers (35.0%) was significantly higher than in male carriers (11.5%) and the aneuploid blastocyst rate in female carriers (13.5%) was significantly lower than that in male carriers (30.8%). In addition, the proportion of ICE in female carriers (41.7%) was significantly lower than in male carriers (71.8%).

There was no significant difference in the unbalanced blastocysts and aneuploid blastocysts rate between RecT carriers and RobT carriers. However, the balanced blastocyst rate in RecT carriers (16.3%) was significantly lower than in RobT carriers (39.5%). In addition, the unbalanced + aneuploid blastocysts rate and proportion of total ICE in RecT carriers (36.2% and 56.6%) were significantly higher than that in RobT carriers (16.3% and 30.2%). Furthermore, in Inv carrier, it was all balanced blastocysts.

### 3.7 Chromosomal abnormality analysis for structural rearrangement in blastocysts of RecT carriers

Table 7 shows the analysis of the meiotic segregation modes in the blastocysts from RecT carriers. Overall, in the unbalanced blastocysts, the incidence of adjacent‐1 segregation was 58.9%, adjacent‐2 segregation was 25.8%, and 3:1 segregation was 15.3%. First, we evaluated the effect of the ovarian stimulation protocols on the meiotic segregation modes and there was no significant difference between the three stimulation cycles. Next, the influence of female age at oocyte retrievals on the meiotic segregation modes was evaluated. There was no significant difference in the incidence of adjacent‐2 segregation and 3:1 segregation among the 3 female age groups at oocyte retrievals. Interestingly, there was a significant difference in the incidence of adjacent‐1 segregation between <35 years (52.2%) and ≧40 years group (76.2%), but there was no difference in 35–39 years groups (57.9%). Then, we evaluated the effect of the carrier's gender on the meiotic segregation modes and found no significant differences.

**TABLE 7:** Analysis of meiotic outcomes for biopsied blastocysts in preimplantation genetic testing for structural rearrangements (PGT‐SR) cycles of Rec carriers

	Number of unbalanced blastocysts	Unbalanced blastocyst rate (%) (/Total unbalanced blastocysts)		
		Adjacent‐1	Adjacent‐2	3:1
Total	124	58.9 (73/124)	25.8 (32/124)	15.3 (19/124)
Stimulation method				
GnRH agonist	61	57.4 (35/61)	24.6 (15/61)	18.0 (11/61)
GnRH antagonist	52	59.6 (31/52)	26.9 (14/52)	13.5 (7/52)
Mild stimulation	11	63.6 (7/11)	27.3 (3/11)	9.1 (1/11)
Female age at OR				
<35	46	52.2 (24/46)	32.6 (15/46)	15.2 (7/46)
35–39	57	57.9 (33/57)	22.8 (13/57)	19.3 (11/57)
≧40	21	76.2 (16/21)^b^	19.1 (4/21)	4.8 (1/21)
Carrier gender				
Female	83	54.2 (45/83)	27.7 (23/83)	18.1 (15/83)
Male	41	68.3 (28/41)	22.0 (9/41)	9.8 (4/41)

*Note*: Different letters indicate significant differences (*p* < 0.05).

Abbreviations: GnRH, gonadotropin‐releasing hormone; OR, oocyte retrieval.

## 4 DISCUSSION

In the present study, to evaluate the efficacy of PGT‐SR by aCGH or NGS for preventing recurrent miscarriages in balanced translocation carriers, we did a retrospective analysis of the chromosomal status of the embryos that underwent PGT‐SR. Subsequently, we analyzed the clinical outcomes of the frozen‐thawed embryo transfer cycles of those embryos (Table 1). We performed 68 PGT‐SR cycles on 31 balanced translocation patients over a period of 8 years. Biopsies were performed on 242 blastocysts (65.4%), out of which 241 were WGA successful blastocysts (99.6%). Moreover, 51 were genetically transferable (21.2%), which was in agreement with the previous studies that showed about 20%–30% of genetically transferable blastocysts.^19^, ^20^, ^21^, ^22^ However, the genetically transplantable embryo rate in PGT‐SR by aCGH or NGS using TE cells in this study was higher than that of FISH using blastomeres on day 3 (10%–20%).^8^, ^30^, ^31^ It is hypothesized that blastomeres with chromosomal abnormalities are naturally eliminated during the development of blastocysts.^32^ In recent years, this hypothesis has also been confirmed by observations of embryos using the time‐lapse system, and blastomeres with unbalanced chromosomes have been reported to be a factor in developmental delay.^33^

In this study, all of the cycles in which clinical pregnancy was confirmed were ongoing pregnancies and no cases of miscarriage were found. Thirty five genetically transplantable blastocysts were transferred into the uterine cavity and the clinical pregnancy rate was 57.1% (Table 1). This result was similar to other NGS‐based PGT‐SR results. However, it was higher than the FISH‐based PGT‐SR,^20^, ^27^, ^34^ which had a clinical pregnancy rate of about 40%.^19^, ^20^, ^21^, ^22^ We assume that the decrease in clinical pregnancy rate in FISH‐based PGT‐SRs could be due to the fact that most of them are done at day 3 embryonic stage, while aCGH and NGS‐based PGT‐SRs are done at the blastocyst stage. In addition, compared with aCGH and NGS, which can comprehensively analyze all the 24 chromosomes, FISH is done with a few types of probes corresponding to the translocated chromosomes only.^14^, ^15^, ^16^, ^17^ Thus, chromosomal abnormalities related to nontranslocated chromosomes cannot be detected in FISH and FISH‐based PGT‐SR analyzed embryos are considered to have a low pregnancy rate. From the above, we can conclude that PGT‐SR with aCGH or NGS in balanced translocation carriers is an effective method for selecting genetically transferable blastocysts to prevent miscarriages. In addition, it is thought that avoiding miscarriage will shorten the time to obtain a baby and reduce physical and psychological distress. However, the continued pregnancy rate per patient who underwent PGT‐SR was 57.1% (Table 1). This result is comparable to the live birth rate with natural conception in the reciprocal translocation carriers.^3^, ^10^ Moreover, there were cases in which genetically transferable blastocysts could not be obtained, and some patients terminated treatment without embryo transfer. Therefore, it is probable that there was no significant improvement in the live birth rates in this study.

Regarding the factors that influence chromosomal abnormalities in blastocysts, we analyzed ovarian stimulation, female age, carrier gender, and types of balanced translocations. In the present study, the optimal ovarian stimulation protocol for producing good quality blastocysts suitable for PGT‐SR could not be clearly shown. There was no significant difference in the number of genetically transferable blastocysts between the GnRH agonist and GnRH antagonist protocols (Table 2). Moreover, there was no significant difference between these two groups regarding ICE in which translocated chromosomes affect the meiosis segregation of nontranslocated chromosomes (Table 6). Therefore, it is thought that differences in ovarian stimulation protocols might not affect the incident rate of aneuploidy in the blastocysts. It has also been shown that there is an increase in the incident rate of aneuploidy in the blastocysts when the total Gn dosage is low.^35^ However, in the present study, there was no significant difference in the initiation and total dosage of Gn between these two ovarian stimulation protocols (Table S2). Therefore, there was no difference in the genetically transferable blastocyst rates between them.

On the contrary, the genetically transferable blastocyst rates were significantly reduced in the mild stimulation with a low Gn dosage (Tables 2 and S2), which is considered to be due to the increase in female age at oocyte retrievals.^36^ Regarding the factors that influence chromosomal abnormalities, in the female age at oocyte retrieval, genetically transferable blastocysts could not be obtained in ≧40 years group compared with the younger age groups (<35 years and 35–39 years; Table 3). Although the unbalanced blastocyst rate was lower in the≧40 years group, the sum of unbalanced blastocyst and unbalanced + aneuploid blastocyst did not differ between the three groups (60.0%, 58.5%, and 63.6; Table 6), as others have reported.^28^, ^37^ However, the aneuploid blastocyst and total ICE rate in ≧40 years group were higher than in the younger age groups. From the above, the results suggest that female age is a significant factor affecting chromosome abnormalities.

Carrier gender may affect chromosome segregation during meiosis, with different segregation patterns reported in females and males.^30^, ^38^, ^39^ In the present study, the genetically transferable blastocyst rates were about 20% for both female and male carriers (Table 4), in agreement with other reports.^30^, ^40^, ^41^ Although the unbalanced blastocysts in male carriers were significantly fewer than in female carriers, there was no difference in the unbalanced blastocyst rates, including Unbalanced + Aneuploid blastocysts (Table 6). Though these results agreed with the previous studies, the aneuploidy of nontranslocated chromosomes did not match.^40^, ^42^ Previous reports have also shown that the carrier gender does not affect the aneuploidy in nontranslocated chromosomes.^30^, ^38^, ^39^ In this study, the average female age at the oocyte retrieval (36.9) was higher than in previous studies (30 ~ 32).^39^, ^42^ Therefore, it was considered that the aneuploid blastocyst rate increased due to the increase in chromosomal abnormalities due to the increase in female age. However, aneuploidy of nontranslocated chromosomes was significantly increased in male carriers in the present study. One of the factors could be the low rate of morphologically good blastocyst formation in male carriers. Chromosomal aneuploidy is associated with blastocyst evaluation and morphologically poor blastocysts have been shown to have high rates of aneuploidy.^43^, ^44^ In the report of embryo observation using the time‐lapse system, embryos with delayed development have shown a high aneuploidy rate.^45^, ^46^ Therefore, it was thought that the aneuploid blastocysts rate was higher in male carriers. However, since the number of male carriers is smaller than that of female carriers, a greater number of cases are needed for further study.

Furthermore, Regarding the factors that influence chromosomal abnormalities in blastocysts, we analyzed types of balanced translocations. First, the mean number of retrieved COCs in RecT carriers (13.1) was lower than in RobT carriers (19.0). It is probably due to the fact that 1/3 of the RecT patients were over 40 years old, so they often choose a cycle with milder stimulation. Moreover, it was thought that the difference in the number of retrieved oocytes was due to the fact that the blood E2 concentration on the day of hCG administration was higher in RobT (4489.6 pg/ml) than in RecT (3381.8 pg/ml). Also, the gametes of translocation carriers produce various patterns of chromosomes during meiosis‐I.^47^ In RecT carriers, the quadrivalent chromosomes are segregated by both alternate and nonalternate modes to theoretically produce 16 different gametes and only alternate segregation results in normal or balanced gametes (2/16). Whereas the segregation patterns in meiotic divisions of RobT carriers that generally involve translocation in the acrocentric chromosomes, theoretically produce 1/6 normal and 1/6 balanced gametes by alternate segregation only. Furthermore, it is shown that RobT carriers have a higher proportion of euploid embryos than Rec carriers.^28^, ^40^ The present study also showed that RobT carriers had a significantly higher proportion of balanced blastocysts than RecT carriers (Table 6), supporting the expected segregation pattern in RecT and RobT carriers. In addition, RecT carriers had more abnormalities in nontranslocated chromosomes and particularly, the proportion of Unbalanced + Aneuploid blastocysts was significantly higher (Table 6). Some reports focusing on ICE show that RecT carriers have a higher abnormality in nontranslocated chromosomes containing unbalanced chromosomes.^27^, ^40^ In addition, the incidence of ICE by the location of breakpoints on the RecT chromosomes has been reported to be high in short‐arm translocations.^26^ The difference in the location of breakpoints in RecT would affect the rearrangement and pairing of translocated chromosomes and normal homologous chromosomes compared with RobT.

Ovarian stimulation protocol, female age at oocyte retrieval and carrier's gender were the parameters for the analysis of the segregation modes of reciprocal translocation. (Table 7). We observed no difference in the segregation pattern in any of the ovarian stimulation protocols. In addition, there was no difference in the segregation pattern due to the carrier's gender, which corresponded with previous reports.^25^ On the other hand, in the female age at oocyte retrieval, there was a significant increase in the incidence of adjacent‐1 segregation in ≥40 years group compared with the 35 years group, but there was no difference was in the 35–39 years group. Moreover, we also compared whether the translocation chromosome contained an acrocentric chromosome (Table S3), the translocation breakpoint was on the short arm or the long arm (Table S4) and terminal breakpoints (Table S5) among the 3 female age groups. As a result, there were no differences in the acrocentric chromosomes, the chromosome arms with breakpoints and the terminal breakpoints, and no association was found with female age at oocyte retrieval. Adjacent‐1 segregation separates chromosomes with nonhomologous centromeres and adjacent‐2 segregation separates chromosomes with homologous centromeres during meiosis in a reciprocal translocation heterozygote. In meiosis, sister chromatids and their centromeres are initially bound by cohesin. In anaphase I, cohesin on the sister chromatid arms is degraded, but around the centromere is not. However, it has been reported that cohesin decreases with age.^48^, ^49^ Therefore, we suspected that as the binding of the centromere region could not be maintained by cohesin, the adjacent‐1 segregation increased due to the separation of the sister chromatid.

Here, for the first time in Japan, we present the clinical results of PGT‐SR by aCGH or NGS, which enables a comprehensive analysis of chromosomes. Notably, the transfer of normal/balanced blastocysts did not result in any miscarriages in this study. Therefore, it can be assumed that PGT‐SR by aCGH/NGS is effective in selecting embryos with chromosomal normality thus preventing recurrent miscarriages. However, the genetically transferable embryos cannot be obtained in female translocation carriers of ≧40 years, and PGT‐SR is considered to be few effective in leading to live birth. Therefore, it is considered that sufficient explanation is necessary for the implementation of PGT‐SR in ≧40 years old. Furthermore, our results may help predict the segregation pattern by comparing various factors leading to better genetic counseling of balanced translocation carriers for PGT‐SR cycles using blastocyst biopsy.

## CONFLICT OF INTEREST

The authors declare no conflicts of interest associated with this manuscript. All procedures followed were in accordance with the ethical standards of the concerned institutional and national committees for human experimentation and with the Helsinki Declaration of 1964 and its later amendments. Informed consent was obtained from all patients for being included in the study. The protocol for the research project including human subjects has been approved by the in‐house ethics board of IVF Namba Clinic.

## Supporting information


Tables S1‐S5
