# Supplementary material for: Analysis of clinical outcomes and meiotic segregation modes following preimplantation genetic testing for structural rearrangements using aCGH/NGS in couples with balanced chromosome rearrangement
Source: Reprod Med Biol. 2022 Jun 29;21(1):e12476. doi: 10.1002/rmb2.12476 (PMC9243298; doi:10.1002/rmb2.12476)
Supplement: Supplementary file 1 — Tables S1‐S5 [file RMB2-21-e12476-s001.docx]

**Table S1. The detailed information for each case included karyotype, past pregnancy history, PGT-SR results and embryo transfer results.**

|  | Carrier’s Karyotype | Other causes of recurrent miscarriage | Past pregnancy history | | | Result of PGT-SR | | | | | | Result of embryo transfer | | |
| --- | --- | --- | --- | --- | --- | --- | --- | --- | --- | --- | --- | --- | --- | --- |
|  |  |  | Number of  pregnancies | Number of  miscarriages | Number of  live births | Average of female age at OR | Number of  cycles | Number of  COCs | Fertilized  oocytes | Biopsied  blastocysts | Genetically  transferable  blastocysts | Number of  ET cycles | Clinical  pregnancies | Ongoing  pregnancies |
| 1 | 46,XX,t(1;16)(p34.3;p12) | - | 3 | 2 | 1 | 39.2 | 3 | 30 | 21 | 7 | 3 | 2 | 1 | 1 |
| 2 | 46,XY,t(15;22)(q21.2;q11.2) | - | 4 | 3 | 1 | 40.8 | 2 | 14 | 12 | 4 | 0 | - | - | - |
| 3 | 46,XY,t(4;5)(q21;q35) | - | 2 | 2 | 0 | 37.5 | 3 | 16 | 11 | 5 | 2 | 2 | 0 | 0 |
| 4 | 46,XX,t(7;8)(q21;p22) | - | 4 | 4 | 0 | 40.0 | 1 | 8 | 4 | 3 | 0 | - | - | - |
| 5 | 46,XY,t(6;14)(p21.3;q32.3) | - | 5 | 5 | 0 | 33.8 | 2 | 42 | 27 | 11 | 2 | 2 | 2 | 2 |
| 6 | 46,XX,t(17;20)(p13;q11.2) | - | 2 | 2 | 0 | 34.3 | 3 | 13 | 9 | 3 | 1 | 1 | 1 | 1 |
| 7 | 46,XX,t(10;18)(p10;p10) | APS | 2 | 2 | 0 | 37.3 | 2 | 53 | 37 | 14 | 2 | 2 | 2 | 2 |
| 8 | 46,XY,t(5;14)(p13.3;p24.1) | - | 3 | 3 | 0 | 34.0 | 3 | 114 | 55 | 5 | 1 | 1 | 1 | 1 |
| 9 | 45,XY,der(14;21)(q10;q10) | - | 2 | 2 | 0 | 39.0 | 1 | 20 | 12 | 7 | 6 | 2 | 2 | 2 |
| 10 | 46,XX,t(7;13)(q33;q22) | - | 3 | 3 | 0 | 38.0 | 1 | 31 | 20 | 4 | 1 | 1 | 1 | 1 |
| 11 | 46,XX,t(2;4)(q22;q23) | - | 2 | 2 | 0 | 34.6 | 4 | 70 | 53 | 30 | 3 | 1 | 1 | 1 |
| 12 | 46,XY,t(4;13)(q23;q22) | - | 3 | 3 | 0 | 30.2 | 4 | 73 | 29 | 4 | 1 | 1 | 0 | 0 |
| 13 | 46,XX,t(9;13)(q33;q12.1) | APS | 4 | 4 | 0 | 37.0 | 1 | 21 | 18 | 8 | 1 | 1 | 1 | 1 |
| 14 | 45XX,der(13;14)(q10;q10) | - | 5 | 5 | 0 | 36.9 | 2 | 43 | 36 | 21 | 6 | 4 | 2 | 2 |
| 15 | 46,XX,t(1;16)(q21;q12.1) | - | 5 | 5 | 0 | 36.6 | 2 | 23 | 21 | 5 | 2 | 2 | 1 | 1 |
| 16 | 46,XX,t(3;17)(q27;q21.3) | - | 2 | 2 | 0 | 32.0 | 3 | 15 | 8 | 0 | - | - | - | - |
| 17 | 46,XX,t(6;7)(q12;q21.2) | - | 3 | 3 | 0 | 45.8 | 7 | 17 | 11 | 3 | 0 | - | - | - |
| 18 | 46,XX,t(11;22)(q23.3;q11.2) | - | 3 | 3 | 0 | 37.2 | 2 | 20 | 13 | 9 | 3 | 2 | 2 | 2 |
| 19 | 45,XX,dic(13;14)(p11.2;p11.2) | - | 3 | 3 | 0 | 31.0 | 1 | 17 | 11 | 8 | 3 | 2 | 1 | 1 |
| 20 | 46,XY,t(1;6)(p32.3;q27) | - | 2 | 2 | 0 | 36.0 | 1 | 30 | 11 | 2 | 0 | - | - | - |
| 21 | 45,XX,der(14;22)(q10;q10) | - | 3 | 3 | 0 | 34.0 | 1 | 15 | 13 | 7 | 2 | 2 | 0 | 0 |
| 22 | 46,XY,t(6;7)(q21;q32) | - | 2 | 2 | 0 | 40.6 | 7 | 73 | 59 | 29 | 0 | - | - | - |
| 23 | 46,XX,der(5)(12qter→12q13.3::5p15.1→5qter),  der(12)(12pter→12q12::12q13.3→12q12::5p15.1→5pter) | - | 3 | 3 | 0 | 43.4 | 2 | 6 | 6 | 2 | 0 | - | - | - |
| 24 | 45,XX,inv(6)(p23q15) | - | 3 | 3 | 0 | 38.0 | 1 | 12 | 8 | 2 | 2 | 2 | 0 | 0 |
| 25 | 46,XX,t(4;6)(q25;p11.2） | APS | 2 | 2 | 0 | 36.0 | 1 | 17 | 11 | 4 | 1 | 1 | 1 | 1 |
| 26 | 46,XX,t(2;17)(p25.3;q25.3) | - | 5 | 4 | 1 | 38.0 | 1 | 12 | 7 | 6 | 1 | 1 | 1 | 1 |
| 27 | 46,XX,t(2;8)(p23;q24.3) | - | 7 | 5 | 2 | 34.0 | 1 | 9 | 7 | 5 | 2 | 2 | 1 | 1 |
| 28 | 46,XY,t(3;13)(q24;q21.3) | - | 2 | 2 | 0 | 30.0 | 2 | 31 | 19 | 11 | 1 | 1 | 1 | 1 |
| 29 | 46,XX,t(2;11)(q24.3;q21) | - | 2 | 2 | 0 | 32.0 | 1 | 32 | 17 | 9 | 0 | - | - | - |
| 30 | 46,XX,t(7;8)(q22;p11.2) | - | 2 | 2 | 0 | 35.0 | 1 | 16 | 12 | 9 | 4 | 3 | 1 | 1 |
| 31 | 46,XX,t(12;13)(p12;q21) | - | 3 | 3 | 0 | 28.2 | 2 | 28 | 13 | 5 | 1 | 1 | 1 | 1 |

OR, Oocyte retrieval; COCs, Cumulus-Oocyte Complex; ET, Embryo transfer; APS, Antiphospholipid syndrome.

**Table S2. The Stimulation parameters of the three stimulation protocols.**

|  | Total | GnRH agonist | GnRH antagonist | Mild stimulation |
| --- | --- | --- | --- | --- |
| Serum anti-Mullerian hormone level  (ng/ml) | 3.2 ± 2.0 | 3.8 ± 2.0^a^ | 3.6 ± 1.8^a^ | 1.1 ± 1.0^b^ |
| Antral follicle counts | 9.9 ± 4.9 | 12.5 ± 4.4^a^ | 11.5 ± 3.4^a^ | 4.5 ± 2.2^b^ |
| Initiation gonadotropin (IU) | 310.7 ± 106.2 | 352.2 ± 81.7^a^ | 358.7 ± 82.0^a^ | 189.7 ± 60.0^b^ |
| Total gonadotropin dosage (IU) | 1888.5 ± 1078.6 | 2340.2 ± 834.7^a^ | 2259.8 ± 782.4^a^ | 770.5 ± 999.5^b^ |
| E2 at hCG day (pg/ml) | 3311.0 ± 2705.6 | 4241.4 ± 2145.6^a^ | 4286.7 ± 3119.3^a^ | 1003.3 ± 879.0^b^ |
| Stimulation length (days) | 12.0 ± 1.8 | 12.0 ± 1.4 | 11.4 ± 1.3 | 12.6 ± 2.3 |

Different letters indicate significant differences (*P* < 0.05).

GnRH, gonadotropin-releasing hormone; E2, Estradiol 2; hCG, Human chorionic gonadotropin.

Table S3. The detailed analysis of meiotic segregation patterns in reciprocal translocation carriers according with or without acrocentric chromosomes.

|  | Average of  female age (years) | Balanced  blastocyst rate (%)  (/Analyzed blastocysts) | Unbalanced blastocyst rate (%)  (/Total unbalanced blastocysts) | | |
| --- | --- | --- | --- | --- | --- |
|  |  |  | Adjacent-1 | Adjacent-2 | 3:1 |
| Non-involved  acrocentric chromosome | 37.1 | 15.4  (22/143) | 62.4  (58/93) | 25.8  (24/93) | 11.8  (11/93) |
| Involved  acrocentric chromosome | 33.6 | 18.9  (10/53) | 48.4  (15/31) | 25.8  (8/31) | 25.8  (8/31) |
| Non-involved acrocentric chromosome | | |  |  |  |
| ＜35 | 33.4 | 12.8  (5/39) | 50.0  (14/28) | 35.7  (10/28) | 14.3  (4/28) |
| 35-39 | 37.3 | 22.7  (17/75) | 62.5  (30/48) | 25.0  (12/48) | 12.5  (6/48) |
| ≧40 | 41.3 | 0.0  (0/29) | 82.4  (14/17) | 11.8  (2/17) | 5.9  (1/17) |
| Involved acrocentric chromosome | | |  |  |  |
| ＜35 | 31.4 | 16.7  (6/36) | 55.6  (10/18) | 27.8  (5/18) | 16.7  (3/18) |
| 35-39 | 37.5 | 30.8  (4/13) | 33.3  (3/9) | 11.1  (1/9) | 55.6  (5/9) |
| ≧40 | 40.8 | 0.0  (0/4) | 50.0  (2/4) | 50.0  (2/4) | 0.0  (0/4) |

Table S4. The detailed analysis of meiotic segregation patterns in reciprocal translocation carriers according to the chromosome arm containing breakpoints.

|  | Average of  female age (years) | Balanced  blastocyst rate (%)  (/Analyzed blastocysts) | Unbalanced blastocyst rate (%)  (/Total unbalanced blastocysts) | | |
| --- | --- | --- | --- | --- | --- |
|  |  |  | Adjacent-1 | Adjacent-2 | 3:1 |
| Both breakpoints  in the short arm | 37.2 | 23.1  (6/26) | 62.5  (10/16) | 12.5  (2/16) | 25.0  (4/16) |
| Both breakpoints  in the long arm | 36.4 | 11.7  (14/120) | 57.1  (48/84) | 27.4  (23/84) | 15.5  (13/84) |
| A breakpoint  in each the short and long arm | 35.0 | 24.0  (12/50) | 62.5  (15/24) | 29.2  (7/24) | 8.3  (2/24) |
| Both breakpoints in the short arm of the chromosomes | | | | | |
| ＜35 | 33.8 | 20.0  (1/5) | 100.0  (2/2) | 0.0  (0/2) | 0.0  (0/2) |
| 35-39 | 38.0 | 23.8  (5/21) | 57.1  (8/14) | 14.3  (2/14) | 28.6  (4/14) |
| ≧40 | － | － | － | － | － |
| Both breakpoints in the long arm of the chromosomes | | | | | |
| ＜35 | 32.2 | 8.7  (4/46) | 48.5  (16/33) | 33.3  (11/33) | 18.2  (6/33) |
| 35-39 | 37.6 | 21.7  (10/46) | 56.3  (18/32) | 25.0  (8/32) | 18.8  (6/32) |
| ≧40 | 41.2 | 0.0  (0/28) | 73.7  (14/19) | 21.1  (4/19) | 5.3  (1/19) |
| A breakpoint in each the short arm and long arm | | | | | |
| ＜35 | 32.7 | 25.0  (6/24) | 54.5  (6/11) | 36.4  (4/11) | 9.1  (1/11) |
| 35-39 | 36.2 | 28.6  (6/21) | 63.6  (7/11) | 27.3  (3/11) | 9.1  (1/11) |
| ≧40 | 41.4 | 0.0  (0/5) | 100.0  (2/2) | 0.0  (0/2) | 0.0  (0/2) |

Table S5. The detailed analysis of meiotic segregation patterns in reciprocal translocation carriers according with or without terminal breakpoints

|  | Average of  female age (years) | Balanced  blastocyst rate (%)  (/Analyzed blastocysts) | Unbalanced blastocyst rate (%)  (/Total unbalanced blastocysts) | | |
| --- | --- | --- | --- | --- | --- |
|  |  |  | Adjacent-1 | Adjacent-2 | 3:1 |
| Non-involved  terminal breakpoints | 35.9 | 12.4  (18/145) | 58.3  (56/96) | 29.2  (28/96) | 12.5  (12/96) |
| Involved  terminal breakpoints | 36.8 | 27.5  (14/51) | 60.7  (17/28) | 14.3  (4/28) | 25.0  (7/28) |
| Non-involved terminal breakpoints | | |  |  |  |
| ＜35 | 32.1 | 11.9  (7/59) | 51.4  (19/37) | 32.4  (12/37) | 16.2  (6/37) |
| 35-39 | 37.1 | 20.0  (11/55) | 56.4  (22/39) | 30.8  (12/39) | 12.8  (5/39) |
| ≧40 | 41.1 | 0.0  (0/31) | 75.0  (15/20) | 20.0  (4/20) | 5.0  (1/20) |
| Involved terminal breakpoints | | |  |  |  |
| ＜35 | 33.8 | 25.0  (4/16) | 55.6  (5/9) | 33.3  (3/9) | 11.1  (1/9) |
| 35-39 | 37.9 | 30.3  (10/33) | 61.1  (11/18) | 5.6  (1/18) | 33.3  (6/18) |
| ≧40 | 43.4 | 0.0  (0/2) | 100.0  (1/1) | 0.0  (0/1) | 0.0  (0/1) |

A translocation with terminal breakpoints was defined as a translocation when at least one of the two translocated segments /arms involved in the translocation had a length ratio of < 0.2.
